# Sequence Analysis of microRNAs Encoded by Simian Lymphocryptoviruses

**DOI:** 10.3390/v16121923

**Published:** 2024-12-16

**Authors:** Yan Chen, Devin N. Fachko, Helen L. Wu, Jonah B. Sacha, Rebecca L. Skalsky

**Affiliations:** 1 Vaccine and Gene Therapy Institute, Oregon Health and Science University, Beaverton, OR 97006, USA; yanc@ohsu.edu (Y.C.); fachko@ohsu.edu (D.N.F.); sacha@ohsu.edu (J.B.S.); 2 Oregon National Primate Research Center, Beaverton, OR 97006, USA; wuhe@ohsu.edu

**Keywords:** lymphocryptovirus, herpesvirus, microRNAs, biomarkers

## Abstract

Lymphocryptoviruses (LCVs) are ubiquitous gamma-herpesviruses that establish life-long infections in both humans and non-human primates (NHPs). In immunocompromised hosts, LCV infections are commonly associated with B cell disorders and malignancies such as lymphoma. In this study, we evaluated simian LCV-encoded small microRNAs (miRNAs) present in lymphoblastoid cell lines (LCLs) derived from a Mauritian cynomolgus macaque (*Macaca fascicularis*) with cyLCV-associated post-transplant lymphoproliferative disease (PTLD) as well as the viral miRNAs expressed in a baboon (*Papio hamadryas*) LCL that harbors CeHV12. Via sequence comparisons, we further predicted viral miRNAs encoded by LCVs that infect two additional NHP species: stump-tailed macaques (*Macaca arctoides*) and bonobos (*Pan paniscus*). Together, these species represent two arms of the primate phylogeny: Hominoids (*Pan*) and Old-World monkeys (*Macaca*, *Papio*). Through our analysis, we defined sequences for >95 viral miRNAs encoded by these four NHP LCVs. Our study provides the most comprehensive annotation of NHP LCV miRNAs to date, yielding a resource for developing sequence-specific reagents to detect these molecules. Importantly, we further demonstrate that cyLCV miRNAs can be detected in circulation in vivo and have biomarker potential for LCV-related PTLD.

## 1. Introduction

Lymphocryptoviruses (LCVs) are ubiquitous gamma-herpesviruses that establish life-long persistent infections in their primate hosts [1]. As infectious pathogens, LCVs are highly successful at infecting their hosts, likely due to their oral route of transmission combined with the shedding of high viral loads in saliva [1]. Epstein–Barr virus (EBV) represents the human counterpart of the LCV family and, besides infectious mononucleosis, is linked to several lymphoproliferative diseases such as post-transplant lymphoproliferative disorders (PTLD) and malignancies such as Burkitt’s lymphoma (BL), Hodgkin lymphoma, and non-Hodgkin lymphoma [2,3].

Despite the identification of EBV in pediatric BL samples over 60 years ago [2,3], there are still no EBV-specific FDA-approved vaccines or therapies [4]. Preclinical animal models of EBV infection and pathogenesis are necessary for the development of vaccines and virus-specific therapeutics; however, relevant small animal models for EBV are limited, and thus, studies predominantly rely on large animal models such as nonhuman primates (NHPs) in order to fully recapitulate all aspects of EBV infection, tissue tropism, immune responses, pathogenesis, and transmission [1,4,5,6,7,8,9]. Notably, LCVs naturally infect humans and other hominids, including both Old and New World NHPs, and thus, LCV infection in macaques is often used to model EBV pathogenesis [1,9] and accurately evaluate targets for EBV vaccine development [10,11]. Akin to EBV infection, LCVs in immunocompromised macaques are associated with lymphoproliferative diseases such as PTLD, lymphoid hyperplasia, and B cell lymphoma [1,6,7,9,12,13].

In terms of genome structure and organization, LCVs are double-stranded DNA viruses of approximately 160 to 175 kb in size and encode similar open reading frames (ORFs) for lytic and latent proteins with near identical arrangement [1,14,15,16]. In addition to viral proteins, LCVs encode several regulatory non-coding RNAs, such as microRNAs (miRNAs), which are expressed throughout the viral life cycle and act to post-transcriptionally regulate gene expression [17,18,19,20]. While viral miRNA sequences are well documented for rhesus LCV, which infects rhesus macaques (*Macaca mulatta*), miRNAs expressed by other NHP LCVs are not fully annotated [17,18,19,20]. As NHPs represent a critical resource for biomedical research due to their close phylogeny to humans, there remains a continuing need to develop and expand molecular tools that support the evaluation of infectious diseases such as persistent gamma-herpesviruses in these models. 

Thus, our overarching goals for this study were (i) to define the miRNAs encoded by LCVs which infect cynomolgus macaques and other NHP species, representing two of the three arms of the primate phylogeny, and (ii) to determine whether these small viral RNAs can be detected in circulation in vivo. Importantly, our data show that multiple cyLCV miRNAs are detected in plasma in a cynomolgus macaque with PTLD, highlighting the utility of viral miRNAs as potential diagnostic and/or prognostic biomarkers for LCV-associated disease.

## 2. Materials and Methods

*Cell culture*: B cell lines were maintained at 37 °C in a humidified atmosphere with 5% CO_2_ in RPMI-1640 supplemented with 15% fetal bovine serum and 1% penicillin/streptomycin/L-glutamine. CyLCV-positive cyLCLs are derived by the spontaneous outgrowth of cyLCV-infected cells from PBMCs, as well as multiple lymph node (LN) tissues from Mauritian cynomolgus macaque (MCM) 35132, which developed PTLD following hematopoietic stem cell transplantation (HSCT) [7,12]. S594 cells from ATCC (CVCL-E151) are infected with CeHV12 and are originally derived from the spontaneous outgrowth of peripheral blood cells from a baboon [21]. Rhesus LCL 309-98 cells are infected with rhesus LCV (rLCV) strain 8664 [9]. EBV-positive Raji (CCL-86) and EBV-negative BJAB are both human BL cell lines. 

*Viral miRNA sequencing and bioinformatics analysis*: Total RNA was isolated from spontaneous cyLCLs established from PBMCs isolated from MCM 35132. A miRNA sequencing library was generated using the NEBNext small RNA kit (New England Biolabs, Ipswich, MA, USA), according to the manufacturer’s protocol. Sequencing was performed on the Illumina HiSeq 2500 (Novogene, Sacramento, CA, USA). Reads were obtained in fastq format. Data were filtered to exclude short reads <16 nt and low-quality reads using FastQC and aligned to the cyLCV genome (Lymphocryptovirus Macaca/pfe-lcl-E3; NC_055142) using Bowtie2 [22], allowing for up to two mismatches.

Total RNA was isolated from S594 cells using TRIzol (ThermoFisher Scientific, Waltham, MA, USA), according to the manufacturer’s protocol, with minor modifications (95% ethanol wash). Small RNAs were enriched from the total RNA using the miRVana miRNA isolation kit (ThermoFisher Scientific, Waltham, MA, USA) and used to generate Illumina TruSeq small RNA sequencing libraries per the manufacturer’s instructions. Sequencing (50 cycle, SE) was performed on the Illumina HiSeq 2000 platform at the OHSU MPSSR. Sequencing reads were obtained in fastq format, filtered using FastQC, and aligned to the *Papio anubis* genome assembly (Panu 3.0) using Bowtie2 [22], allowing for up to two mismatches. Aligned reads were mapped to known primate pre-miRNA sequences annotated in miRBase v21 using scripts from the miRDeep2 pipeline [23]. Remaining unmapped sequences were initially aligned to the EBV (NC_007605) and rhesus LCV (NC_006146) genomes and then the assembled CeHV12 BART region (see below) with up to two mismatches to identify potential viral miRNAs.

*DNA isolation, PCR amplicons, and sequence analysis of the CeHV12 BART region*: Genomic DNA was isolated from S594 cells using DNAzol (ThermoFisher Scientific, Waltham, MA, USA), according to the manufacturer’s protocol. Primers for the PCR amplification of the CeHV12 BART region and sequencing are listed in Table S2. PCR products were amplified from S594 genomic DNA, purified by electrophoresis on 1% tris-acetate EDTA (TAE) agarose gels, and submitted for Sanger sequencing. The CeHV12 BART Cluster II region was subsequently assembled in Geneious Prime 2023.0.4 from sequenced PCR amplicons, obtaining approximately three-fold coverage. 

*Prediction of Pan paniscus LCV1 and HVMA pre-miRNAs*: To define Pan paniscus LCV1 pre-miRNAs, Pan paniscus isolate Ulindi viral contigs (AJFE01003225.1, AJFE01121077.1, AJFE01006050.1) with significant homology (based on BLAST alignments) to EBV BHRF1 and BART regions were aligned to known EBV and rLCV pre-miRNA sequences using the multiple sequence alignment Clustal Omega (http://www.ebi.ac.uk/Tools/msa/clustalo/) (last accessed on 19 November 2024). Pan paniscus LCV1 regions with extensive homology to LCV miRNA hairpins were extracted and folded with RNAfold (http://rna.tbi.univie.ac.at/cgi-bin/RNAWebSuite/RNAfold.cgi) (last accessed on 19 November 2024) and mFold [24] to define candidate pre-miRNA hairpin structures. To determine HVMA pre-miRNAs, EBV and rLCV pre-miRNA sequences were aligned to the Macaca arctoides gammaherpesvirus 1 genome (MG471437.1, Macacine gammaherpesvirus 13) using Clustal Omega. Regions with extensive homology were extracted and folded with mFold [24] to confirm hairpin structures.

*Plasma miRNA extraction and miRNA qRT-PCR*: miRNAs were isolated from plasma samples as previously described [25]. Briefly, miRNAs were extracted from plasma using the miRNeasy mini kit (Qiagen, Hilden, Germany) and eluted into nuclease-free water. To assay miRNA expression, total RNA was reverse-transcribed using miRNA-specific Taqman (ThermoFisher Scientific, Waltham, MA, USA) primers and miRNAs were detected using miRNA-specific Taqman probes and Taqman Universal PCR mastermix (ThermoFisher Scientific, Waltham, MA, USA). For the detection of miR-cyL-10-5p and miR-cyL-30-5p and the corresponding rLCV miRNAs, the EBV miRNA stem-loop primers and Taqman probes for miR-BART5 and miR-BART20 were used. For the detection of miR-cyL-2-5p, we used stem-loop primers that can amplify both rLCV miR-rL1-2-5p and EBV miR-BHRF1-2-5p and the distinct Taqman probes that are specific for either miR-cyL-2-5p/miR-rL1-2-5p or miR-BHRF1-2-5p. miRNA levels were normalized to miR-16. All PCR reactions were performed in technical replicates.

*Statistical analysis*: Statistical analysis was performed in GraphPad Prism 10. Student’s *t*-test was used to compare miRNA levels between plasma samples, and *p* values of <0.05 were considered significant.

## 3. Results

### 3.1. Identification of Viral miRNAs Encoded by Cynomolgus Macaque Lymphocryptovirus (cyLCV) 

The genome of cyLCV was first sequenced in 2016 [15], and based on >96% sequence identity following pairwise alignment to the rLCV BHRF1 region, Kampershroer et al. reported four potential cyLCV homologs of the BHRF1 miRNAs. Interestingly, regions of the cyLCV genome with no predicted protein coding sequences, such as ~123–133 kbp and ~149–153 kbp, which flank cyLCV LF3 where other LCV BART miRNAs are known to be encoded, exhibited high levels of dissimilarity to rLCV [15] and thus, no other cyLCV miRNAs were annotated at that time. Recently, we reported five new genome sequences for cyLCVs isolated from multiple MCM with PTLD or PTLD-like disease [7]; these strains exhibited ~99.7% identity within their predicted BART-miRNA encoding regions. The pairwise alignments of these cyLCV isolates (PQ385619, PQ385620, PQ385621, PQ385622, PQ385623) to corresponding BART regions of the rhesus LCV genome revealed 57.9–60.5% identity, with several smaller ~50–70 nucleotide (nt) regions exhibiting >85% identity, suggesting that multiple BART viral miRNA homologs are indeed encoded by cyLCV.

**Figure 1:**
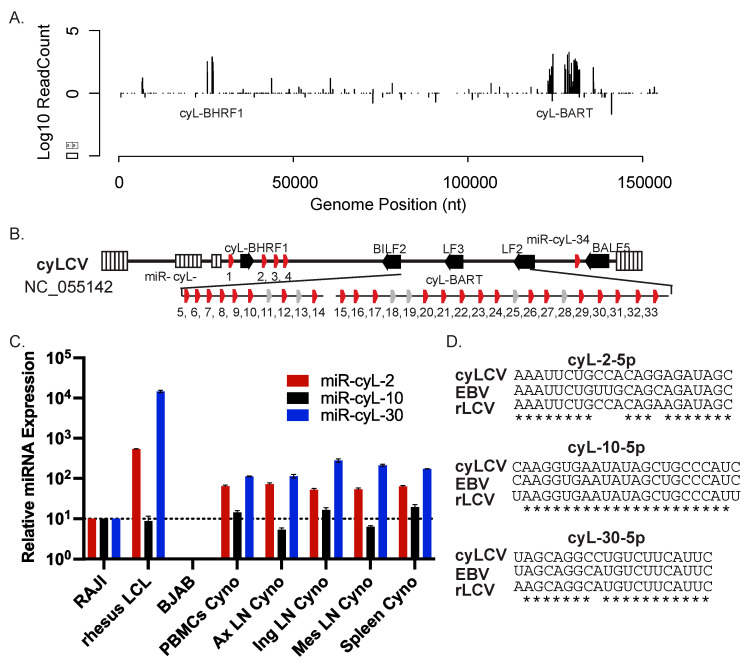
Identification of cyLCV miRNAs. (**A**) Distribution of reads aligned to cyLCV genome. (**B**) Positions of viral pre-miRNAs in the cyLCV genome. Red indicates experimental evidence of miRNA expression; gray indicates a predicted cyLCV pre-miRNA based on homology to the rLCV and EBV pre-miRNAs. (**C**) qRT-PCR analysis of three conserved viral miRNAs in primary cyLCLs established from tissue biopsies from MCM 35132. miRNA expression is shown relative to levels in Raji and rLCL 309-98 cells and normalized to miR-16. PBMCs = cyLCLs sequenced in (**A**); Ax LN = axillary lymph node; Ing LN = inguinal lymph node; Mes LN = mesenteric lymph node. (**D**) Sequence alignments of cyLCV, EBV, and rLCV miRNAs tested by qRT-PCR.

To gain experimental evidence for cyLCV-encoded miRNAs, we analyzed the small RNA population in a cyLCV-infected B cell line (MCM 35132 cyLCLs) that spontaneously outgrew from PBMCs of a MCM presenting with PTLD [7,12]. A small RNA library was generated using the NEB Next kit and sequenced on the Illumina platform. While the total read counts were low (172,160 reads due to sample multiplexing), we observed 5116 reads that mapped directly to the cyLCV genome (Figure 1A). Comparable to previous LCV small RNA sequencing studies, we identified a few reads mapping to the cyLCV EBER regions; however, most viral hits occurred in the BHRF1 and BART homologous regions of the genome, consistent with small RNAs originating from these conserved viral miRNA loci. Following alignments to the cyLCV, rLCV, and EBV genomes, potential pre-miRNA folding structures were determined by mFold [24]. The major cyLCV miRNA sequences with annotated 5′ and 3′ ends are listed in Table S1. In total, we defined 34 distinct pre-miRNA hairpin structures for cyLCV, with direct experimental evidence for 28 of these pre-miRNAs (Figure 1B; Table S1). 

To obtain the further experimental evidence of cyLCV miRNAs, we extracted total RNA from additional cyLCLs that were independently derived from lymph node (LN) and tissues (axillary, inguinal, and mesenteric LN, spleen) collected from MCM 35132 at necropsy [7,12]. Using qRT-PCR, we tested the expression of three highly conserved viral miRNAs: homologs of EBV miR-BHRF1-2-5p (cyLCV miR-cyL-2-5p), EBV miR-BART5 (cyLCV miR-cyL-10-5p), and EBV miR-BART20 (cyLCV miR-cyL-30-5p), which all exhibit 100% homology within their seed regions (nucleotides two through eight) (Figure 1D). EBV-positive Raji cells and rLCV-positive rLCLs were included as controls. Figure 1C shows the expression level for each cyLCV miRNA in cyLCLs. Taken together, these data confirm the presence of cyLCV miRNAs and provide the first annotation of the small RNAs encoded by cyLCV. 

### 3.2. Prediction of miRNAs Encoded by Bonobo Lymphocryptovirus (Pan paniscus LCV1)

**Figure 2:**
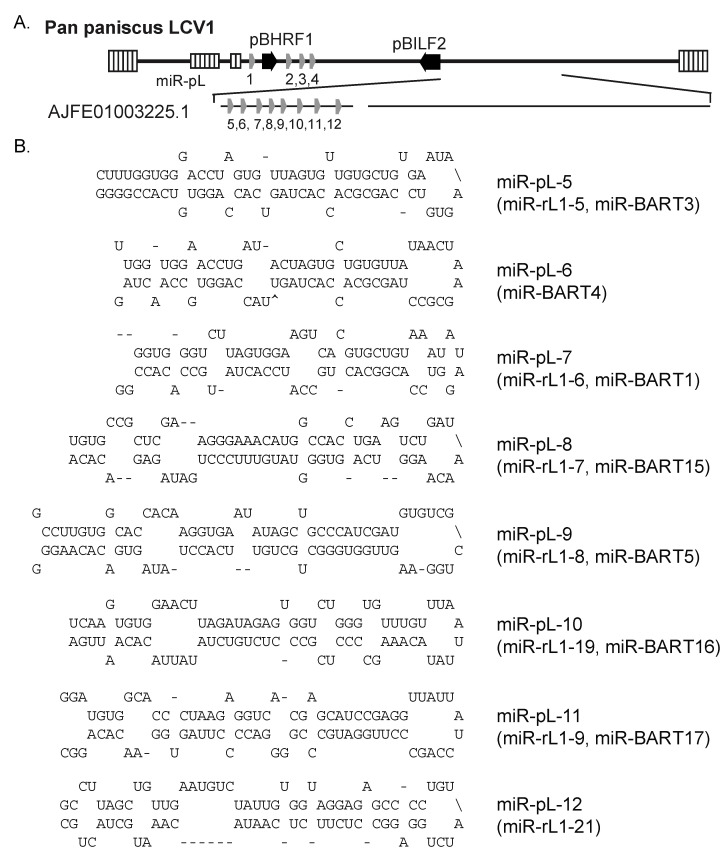
Pan paniscus LCV1 miRNAs. (**A**) Positions of predicted viral miRNAs encoded within the Pan paniscus LCV1 genome. (**B**) Putative hairpin structures for eight miRNA precursors encoded within the conserved BART Cluster I.

In 2014, Aswad and Katzourakis reported novel sequences for Pan paniscus LCV1 following a query of primate genomes in the NCBI whole genome shotgun (WGS) database for herpesvirus-like sequences [26]. Pan paniscus LCV1 naturally infects bonobos (*Pan paniscus*; also referred to as pygmy chimpanzees) and epidemiological studies show the virus is highly disseminated amongst wild bonobos [27]. Notably, three of the reported viral contigs (AJFE01003225.1, AJFE01121077.1, and AJFE01006050.1) overlap the BHRF1 and BART miRNA encoding regions of EBV and other LCVs. To determine whether Pan paniscus LCV1 encodes miRNAs, we therefore aligned EBV, rLCV, and cyLCV pre-miRNA sequences to these three contigs initially using NCBI BLAST and subsequently, identified conserved pre-miRNAs through pairwise alignments in Clustal Omega. Putative Pan paniscus LCV1 miRNA precursors were extracted and folded with mfold [24] to define potential hairpin structures (Figure 2). No BART Cluster II miRNAs could be determined for Pan paniscus LCV1 since the contig sequence AJFE01003225.1 ends within BART Cluster I. In total, we identified 12 conserved hairpins corresponding to homologs of all four BHRF1 miRNA precursors and eight BART Cluster I miRNA precursors. 

### 3.3. Prediction of miRNAs Encoded by HVMA (Macacine Gammaherpesvirus 13)

**Figure 3:**
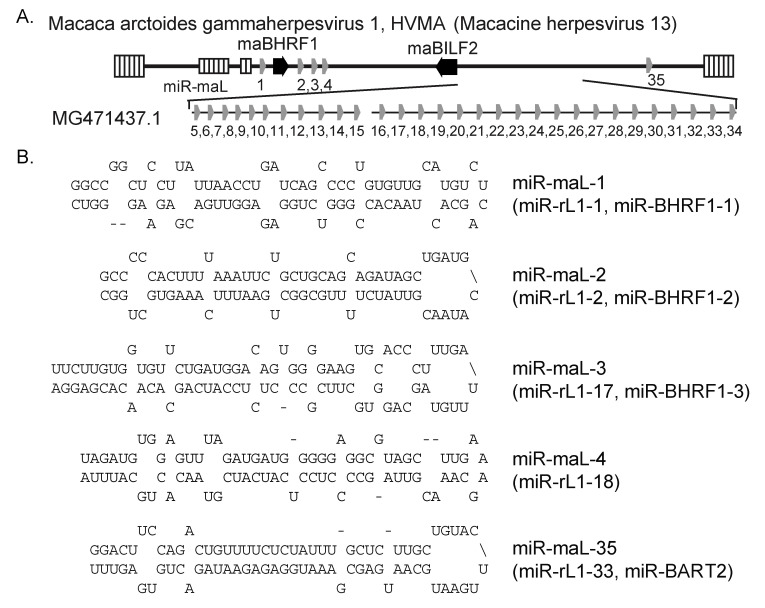
Predicted HVMA miRNAs. (**A**) Positions of predicted viral miRNAs encoded within the HVMA genome. (**B**) Putative pre-miRNA structures for the four BHRF1 miRNA homologs and miR-maL-25 encoded antisense to HVMA BALF5.

The genome of an LCV isolate, HVMA, that naturally infects stump-tailed macaques (*Macaca arctoides*) was recently sequenced [16]. To determine whether HVMA encodes viral miRNAs, we aligned the rLCV and cyLCV pre-miRNA hairpin sequences to the HVMA genome initially using NCBI BLAST and subsequently, identified conserved pre-miRNA sequences using Clustal Omega. Putative HVMA miRNA precursors were extracted and folded with mfold [24] to define the hairpin structures (Figure 3). Comparable to rLCV, cyLCV, and Pan paniscus LCV1, the homologs of four BHRF1 miRNA precursors were present in the genome (Figure 3A). Moreover, we identified 31 putative pre-miRNA structures with homology to other LCV BART miRNAs, including miR-maL-35 which is encoded antisense to the BALF 3′UTR akin to EBV miR-BART2. Predicted pre-miRNA structures for 5 of the 35 predicted HVMA miRNAs are shown in Figure 3B.

### 3.4. Experimental Identification of BHRF1 miRNA Homologs in CeV12-Infected S594 Cells

**Figure 4:**
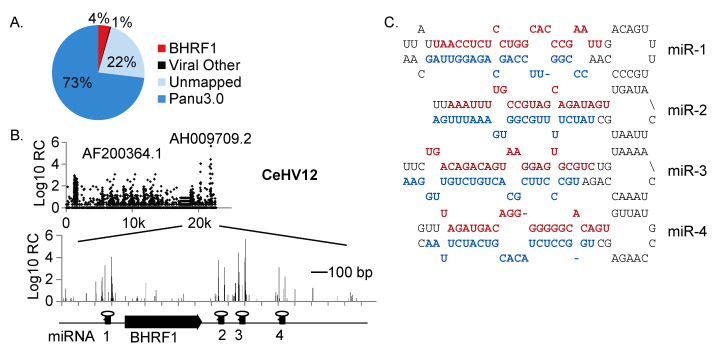
CeHV12 BHRF1 miRNA homologs expressed in S594 cells. (**A**) Distribution of small RNA reads aligned to the baboon genome (Panu3.0) and contigs of the CeVH12 genome. 4% of reads aligned to two CeHV12 contigs that encompass the homologous BHRF1 region (AF200364.1 and AH009709.2). (**B**) Genome plot of small RNA sequencing reads aligned to the CeHV12 BHRF1 region. Y-axis indicates read counts (RC) (**C**) Putative pre-miRNA folding structures and experimentally defined 5p (colored in red) and 3p (colored in blue) sequences of the CeHV12 BHRF1 miRNAs.

To experimentally define the miRNAs of CeHV12, also called Papiine gammaherpesvirus 1, we isolated and sequenced the small RNAs (<200 nt) expressed in S594 cells. S594 cells were originally derived from spleen tissue of a female baboon (*Papio hamadryas*) with lymphoreticular hyperplasia and are persistently infected with CeHV12 (Cercopithecine herpesvirus 12) [21]. Small RNAs were extracted using the miRVana miRNA isolation kit, a cDNA library was prepared using the TruSeq small RNA kit, and miR-Seq (50-cycle, single end) was performed on the Illumina HiSeq2000. We obtained 15,971,595 total reads that were parsed, filtered, and aligned initially to the baboon (*Papio anubis*) genome (Panu3.0). To determine the cellular miRNAs, reads were aligned to all known primate miRNAs present in miRbase v21. Pre-miRNAs and candidates were additionally analyzed by miRDeep2 [23], resulting in the detection of >250 cellular miRNAs, which accounted for 33% of the library (Figure 4A). The most abundant cellular miRNAs included homologs of human miR-148a, miR-92a, miR-146b, miR-21, miR-26a, and miR-191, which is consistent with a B cell miRNA profile. 

In prior studies, four CeHV12 BHRF1 miRNA homologs were predicted [17]. Based on the known locations of other LCV miRNA clusters, we predict that CeHV12 encodes at least twenty other distinct viral pre-miRNAs, beyond the four that are in the BHRF1 region. The remaining viral pre-miRNAs are presumably within the homologous BART region; however, the CeHV12 genome is not yet fully sequenced. To initially map miR-Seq reads of viral origin, we thus downloaded the 15 available CeV12 partial sequences from NCBI, including the H. papio FGARAT and BHRF1 regions (NCBI Accession # AF200364.1 and # AH009709.2). A total of ~4% of reads aligned to the BHRF1 locus, and spanned regions across the individual pre-miRNAs that were previously predicted [17], providing the first experimental evidence for these viral miRNAs (Figure 4B). 

Confirming previous predictions, CeHV12 miR-1 through miR-4 exhibit high sequence conservation to the EBV, rLCV, and cyLCV BHRF1 homologs (Figure 4C, Table S1) [17]. In human and rhesus macaque LCLs, EBV miR-BHRF1-2-3p and rLCV miR-rL1-2 are often the most abundant BHRF1 miRNAs expressed [17,18]. Based on miRNA read counts, we were surprised to find that CeHV12 miR-3-3p, a rLCV miR-rL1-17 and EBV miR-BHRF1-3 homolog, was the most abundant miRNA detected from this region and ranked fourth amongst all miRNAs detected in S594 cells (Figure 4B). Of note, both rLCV miR-rL1-17 and EBV miR-BHRF1-3 target the 3′UTRs of the viral BZLF1 encoding an immediate early (IE) viral transactivator [28]. While there are sequence differences between miR-rL1-17 and CeHV12 miR-3, it is plausible that CeHV12 miR-3 also regulates IE gene expression. Interestingly, both strands of CeHV12 miR-1 were detected, and we observed ten-times higher levels of the 3p strand compared to the 5p (Table S3). This is directly opposite to what is normally seen for the EBV homolog, miR-BHRF1-1, for which the 5p arm is the dominant strand [17]. As LCVs have species-specific tropism [1], it is likely the nucleotide changes within these viral miRNA homologs have arisen to reflect the need for species-specific functions in terms of the target regulation. 

### 3.5. Sequence Alignments Reveal Novel CeV12 BART Cluster II miRNAs

**Figure 5:**
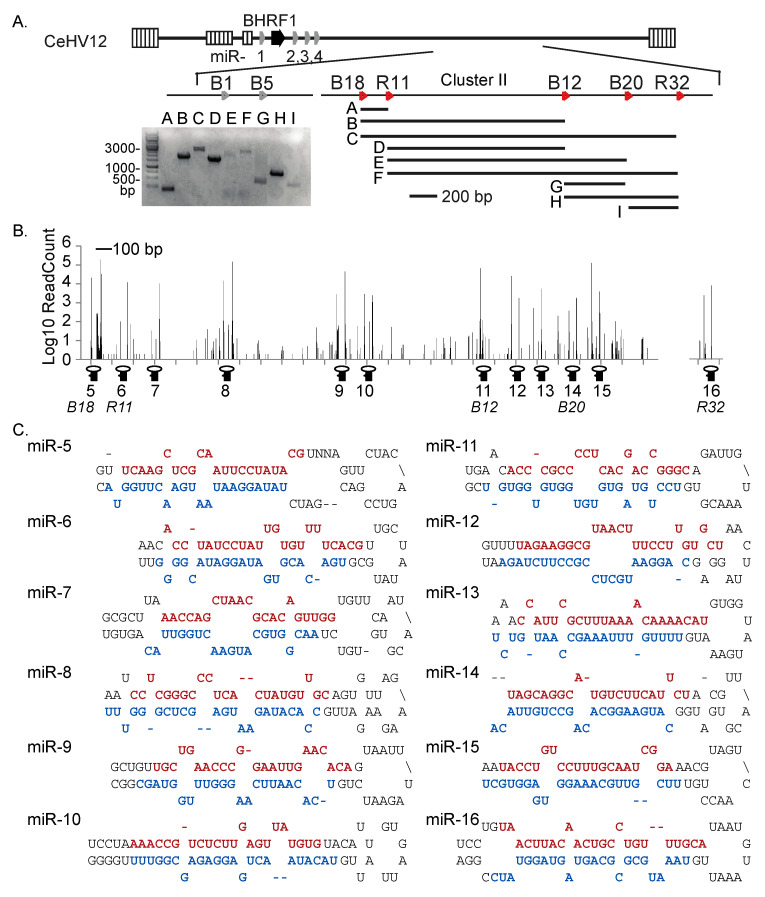
Identification of BART Cluster II miRNA homologs in CeHV12. (**A**) Schematic showing PCR primer positions and regions amplified from S594 genomic DNA. Primers correspond to miRNA sequences conserved with EBV (denoted with B) or rLCV (denoted with R). Insert: agarose gel electrophoresis following PCR amplification of CeHV12 BART regions. (**B**) Genome plot of reads aligned to the CeHV12 BART Cluster II region. (**C**) Predicted pre-miRNA folding structures with the highlighted experimentally defined 5p (colored in red) and 3p (colored in blue) sequences of the CeHV12 BART miRNAs.

Using the genomic information from the homologous BART regions of EBV, rLCV, cyLCV, HVMA, and Pan paniscus LCV1, we next interrogated the S594 miR-Seq library for BART miRNA homologs. Partial homology was observed for sequences matching to multiple pre-miRNAs in EBV BART Cluster II and the homologous rLCV region—specifically EBV miR-BART18, rLCV miR-rL1-11, EBV miR-BART12, EBV miR-BART20, and rLCV miR-rL1-32. We also identified homologs of EBV miR-BART1, miR-BART5, and miR-BART15 in BART Cluster I (Figure 5A; Table S3). Based on the five potential CeHV12 miRNAs with homology to EBV BART Cluster II, we designed several PCR primers to amplify this miRNA-encoding region of the CeHV12 genome (Table S2). Primer combinations were selected based on their predicted, evolutionarily conserved binding positions and used to generate PCR amplicons for Sanger sequencing. The sizes of PCR products are shown in Figure 5A. We were unable to obtain PCR products using the B1/B5 primer pair, and thus, no BART Cluster I miRNA precursors could be determined. Amplicons were gel purified, sequenced, and analyzed using Geneious Prime to compile the CeHV12 BART Cluster II region. This initial round of sequencing provided at least three-fold sequence coverage. Additional PCR amplicons were generated following the first round of sequencing to obtain a near-complete sequence of the CeHV12 BART Cluster II region. 

S594 miR-Seq reads were subsequently aligned to the CeHV12 BART Cluster II region, revealing 12 areas with substantial numbers of mapped reads (Figure 5B). These 12 regions were extracted and folded in mfold [24] to identify miRNA hairpin structures (Figure 5C). A total of 22 mature CeHV12 BART miRNAs arising from the 12 pre-miRNAs were defined. Table S3 lists the major isoforms of the 16 viral miRNAs detected in S594 cells. While no precursor structures are available for any CeHV12 miRNAs that arise from Cluster I, we have also included sequences for three putative viral miRNAs (miR-17, -18, -19) based on their alignments to EBV miR-BART1, miR-BART5 (rLCV miR-rL1-8), and miR-BART15 (rLCV miR-rL1-7) (Table S3). Collectively, this analysis identified 19 CeHV12-encoded pre-miRNAs and defined the distinct 5′ ends for these small RNA molecules.

### 3.6. Circulating cyLCV miRNAs Are Detectable in MCM with PTLD

**Figure 6:**
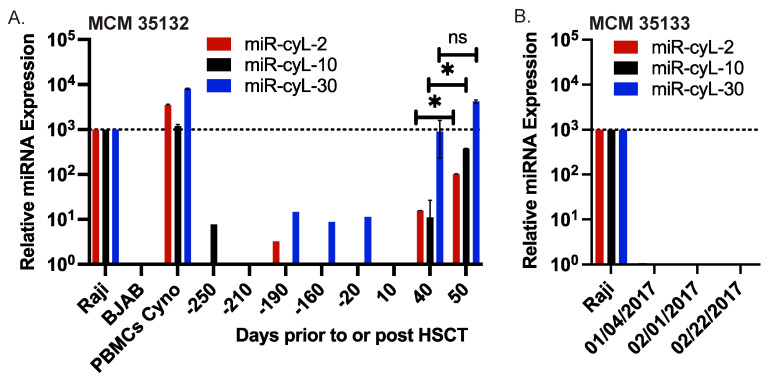
Detection of cyLCV miRNAs in circulation. (**A**) Plasma was collected from MCM 35132 (recipient) at times indicated prior to or post hematopoetic stem cell transplant (HSCT). (**B**) Plasma was collected from MCM 35133 (donor) at three different time points starting ~6.5 months after graft collection. For both (**A**,**B**), miRNAs were extracted from plasma samples and tested by qRT-PCR. Values are normalized to miR-16 and reported relative to the respective EBV miRNA homolog in Raji cells. RNA isolated from rLCL 309-98 and from cyLCL 35132 (spontaneously cultured out of PBMCs) were included as controls. PCR was performed in duplicate. * Student’s *t*-test, *p* < 0.05.

Viral miRNAs have significant promise as diagnostic biomarkers for viral infections and as prognostic biomarkers of viral pathogenesis [29]. Moreover, there is substantial interest in developing non-invasive miRNA-based biomarkers for disease as these RNA molecules are stable in liquid biopsies such as plasma. Using qRT-PCR assays, we tested cyLCV miRNA expression in cell-free plasma samples from MCM 35132, a HSCT recipient that developed aggressive cyLCV-associated PTLD [7,12]. Plasma samples from MCM 35133, the corresponding healthy HSCT donor, were tested in parallel. CyLCV miRNAs were strongly detected at both 40 and 50 days post HSCT in MCM 35132, with significant increases in miR-cyL-2 and miR-cyL-10 between day 40 and 50 (Figure 6A), coinciding with the clinical detection of PTLD. Strikingly, circulating miR-cyL-30 in plasma samples at days 40 and 50 was quite elevated as levels were similar to the cell-associated miR-cyL-30 levels detected in cyLCLs (Figure 6A). Low levels of the cyLCV miRNAs were detected in MCM 35132 prior to HSCT—even at 250 days prior—which indicates pre-existing cyLCV infection (Figure 6A) and is consistent with the previously reported low level of cell-free cyLCV DNA detected in this macaque at day -20 prior to HSCT [12]. No cyLCV miRNAs were detected in plasma samples from the HSCT donor, MCM 35133 (Figure 6B). Such observations suggest that the cyLCV-associated small RNA signature in MCM 35132 likely arose due to the reactivation of the pre-existing cyLCV infection in response to HSCT. Given the abundant level of miR-cyL-30 (homolog of EBV miR-BART20) found in circulation, we anticipate this viral miRNA to be a potential diagnostic marker of cyLCV-associated PTLD and viral pathogenesis in future studies with MCM. 

## 4. Discussion

In this study, we used small RNA-sequencing and in silico sequence alignments to identify conserved viral miRNAs encoded by four different simian LCVs that naturally infect Old World monkeys and hominoids. Importantly, we provide direct experimental support for and document the exact 5′ ends of viral miRNAs encoded by two of these LCVs: cyLCV that infects cynomolgus macaques and CeHV12 that infects baboons. As the 5′ seed sequence of a mature miRNA, which minimally consists of nucleotides two through seven, is a fundamental feature for miRNA function and essential for canonical base pairing with target messenger RNAs [30], the precise annotation of the 5′ end provides key insight into the potential regulatory capabilities for these viral miRNAs.

LCVs exhibit species-specific tropism [1], which presumably arose during co-evolution with their individual primate hosts. From fossil record evidence, the divergence of cercopithecoids (*Papio*, *Macaca*) and hominoids (*Homo*, *Pan*) is estimated to have occurred ~25 million years ago (25 MYA) while divergence of *Papio* and *Macaca* is estimated at 10–12 MYA [31,32]. Interestingly, based on the phylogenetic analysis of the viral DNA polymerase genes (homologs of EBV BALF5) from >45 simian LCVs, two main lineages of LCVs infecting Old World monkeys were proposed [33,34]. Most notably, the divergence of EBV and other LCVs that infect hominids and LCVs that infect cercopithecoids was estimated at 12 MYA [34]. As such, at the genetic level, LCVs are thought to be much closer in evolutionary time compared to their respective host species.

While LCVs are thought to have circulated amongst primates and subsequently co-evolved with their respective host species well after the divergence of hominids and macaques, these viruses exhibit synteny in their genome organization and significant sequence homology has been documented in their protein coding genes [14,15,16,26]. Taking advantage of these genetic attributes enabled us to compare reference viral genomes and published sequence contigs to predict the ~60 nt pre-miRNAs for two other simian LCVs that infect stump-tailed macaques and bonobos. As there is no precise evolutionary model for viral miRNA-encoding genes, we relied on the structural hallmarks of miRNA precursors and sequence identity in the mature miRNA seed regions to define these additional LCV pre-miRNAs. Intriguingly, the multiple sequence alignment of the LCV BART Cluster I regions from EBV, rLCV, cyLCV, HVMA, and Pan paniscus LCV1 revealed only 48.3% identity overall; however, higher sequence homology (>80–88% identity) is observed within the ~60 nt pre-miRNAs themselves, indicating that these small RNA-encoding regions are maintained through ongoing selective pressure. Only minor differences in the miRNA seed regions (nt 2–8) are observed for most NHP LCV BART miRNAs, which likely arose due to species-specific tropisms for these viruses.

Sequence variations in the BART Cluster II region have been reported for EBV type 2 strains, including several strains circulating in Papa New Guinea [35]. Most noteworthy is a ~71–73 nt expansion between EBV miR-BART21 and miR-BART18 [35] that is not observed in EBV type 1 strains and potentially encodes an RNA with a hairpin-like structure. Comparing the BART Cluster II from EBV type 2 (NC_009334) to the BART Cluster II regions of rLCV, cyLCV, and HVMA, we observe homologs of both EBV miR-BART21 and miR-BART18 in the NHP LCVs. However, between the miR-BART21 and miR-BART18 homologs is an additional miRNA; in rLCV, this miRNA is miR-rL1-10 [17,18,20], and in cyLCV, this miRNA is miR-cyL-17. While future studies will be needed to confirm whether the documented ~71–73 nt expansion in EBV type 2 indeed encodes a Drosha substrate that can be processed into functional miRNA(s), it is quite plausible this region represents a positional homolog of the rLCV miR-rL1-10 precursor.

In summary, we successfully annotated >95 viral miRNAs encoded by simian LCVs that naturally infect hominoids and Old-World monkeys. These results provide a molecular resource that enables the development of sequence-specific reagents to track LCV gene expression in vivo. To our knowledge, this is the first study that reports experimental evidence of cyLCV and CeHV12 miRNAs and, notably, demonstrates that cyLCV miRNAs, such as miR-cyL-30, have utility as circulating biomarkers of viral infection in PTLD. Further efforts to sequence additional LCV genomes and miRNAs can strengthen these initial observations and provide tools for new diagnostics of LCV-associated diseases.

## Data Availability

miRNA sequencing datasets can be accessed through the NCBI SRA database under BioProject ID PRJNA1186720.

## Supplementary Materials

The following supporting information can be downloaded at: https://www.mdpi.com/article/10.3390/v16121923/s1. Table S1: cyLCV miRNA sequences; Table S2: Primers used for CeHV12 amplicon sequencing; Table S3: CeHV12 miRNA sequences.
