# Supplementary material for: Sequence Analysis of microRNAs Encoded by Simian Lymphocryptoviruses
Source: Viruses. 2024 Dec 16;16(12):1923. doi: 10.3390/v16121923 (PMC11680086; doi:10.3390/v16121923)
Supplement: Supplementary file 1 [file viruses-16-01923-s001.zip › viruses-3351100-supplementary/Table S1 CynoLCV mIRs.pdf]

Table S1. CyLCV miRNA sequences, MCM35132 LCL-PBMC

Pre-miR	5p	3p	Read Counts (5p, 3p, total)
cL1	TAACCTGATCAGCCCCGGGGTT	CTCCGGGTCTGAAGAGGTTGAC	120, 24, 371
cL2	AAATTCTGCCACAGGAGATAGC	TATCTTTTGCGGGGGAATTT	10, 250, 810
cL3	TGATGGACAGCGGGGAAGTGCACT	TGCTTCGCCCTCTCCATCATA	220, 18, 780
cL4	TAGATGATTGGGGGCGGCGTATCT	TCAGCCCCTCCCCATCATCTTG	63, 15, 345
cL5	CCGTGGGGGCCGTGTGTGCAGT	TCGCACCGCGGCACCCCATGG	7, 1, 12
cL6		CACCACACGTTCCACTGGGTCCAC	0, 2, 5
cL7	ACTTAGTGGTTGGGATGTCCT	*AGCATCCTACCTCACT*	1, 1, 5
cL8	AACCTAGTGCCAGTGATGTGCT	CGCACCACTGTTCACTGGGTCT	2, 19, 92
cL9	AGGGAAATAGCGGCCAACTGAAGTCT	GTCAGTGGGCCTGTTTCCCT	3, 6, 35
cL10	CAAGGTGAATATAGCTGCCCATC	GTGGGCCACTGTTCACCTATA	1, 2, 4
cL11	TAAGAGGACACCGGTACGCCAAGT		31, 0, 124
cL12	TAGATTGTGGAGGAGAGCCCTTT	AGGACTCTTCCCACAATCCCAA	814, 1, 1266
cL13		TTCACTGTATCATCCCCATGATT	0, 68, 189
cL14	TCACTAGTGCTGGCACCA	TTAGTTGTTTGCACTGGTAATT	1, 53, 84
cL15	TAGCTCTCTGTTGACCTTACA	TAAGGTGCAATGAAGAGCTGACT	359, 23, 1215
cL16		GACGGTGCATGAACTGGCTAGA	0, 1079, 1803
cL17	CCTGGACATTCGCTATGAAAC	GATCATAGCTAAAGTCCAGGCT	6, 17, 41
cL18	ACCAGTTCCTGAGATTGTACA	TAACATTCTTTGGGATTGGAGA	2, 148, 242
cL19		TTACAATTTAAAGGTCTAGT	0, 2, 3
cL20	*GCCGGCGCTGGACCC*	TACAATGCCTATGGGTCTAGTAGT	1, 3, 12
cL21	TCGGACAGTCTGGTGCGCCAGTT	ACGCACATCAGGCTGAACGAGC	171, 133, 799
cL22		TCCTGTAGTGAATGGGTGTGGTTT	0, 158, 546
cL23	ACATTCCCTCCAAACAGAATACT	TATTTTGTTTGGCTGGGTATGGA	20, 115, 494
cL24	TAGCAGGCCTGTCTTCATTC	TATGAAGTCATGGCCTGTTG	4, 51, 315
cL25	TCCAAACCTTCGAAGGACGA	TTTCGTTTTTGAAGGTTGGTTGT	6, 77, 163
cL26	TCGCTTACTACTGCCGCATAA	TTATGCGCGCAGTAGAAGCT	3, 23, 94
cL27	TTCCCTACGCCTGCCATTTACG	TAAATGCTAGTAGTAGGGATCT	23, 42, 114
cL28	TATTTTCTCCATTTGCTCTTG	AATGAGCAAAATGGAGAGGAT	16, 37, 171
