# Supplementary material for: Sequence Analysis of microRNAs Encoded by Simian Lymphocryptoviruses
Source: Viruses. 2024 Dec 16;16(12):1923. doi: 10.3390/v16121923 (PMC11680086; doi:10.3390/v16121923)
Supplement: Supplementary file 1 [file viruses-16-01923-s001.zip › viruses-3351100-supplementary/Table S2.pdf]

Table S2. Oligonucleotides for Sequencing BaLCV (CeHV12) BART miRNA region

Name	Sequence
B12rev	ACACCACACCACACACTACAGGA
B12fwd	TCCTGTAGTGTGTGGTGTGGTGT
B20rev	TGTAACAGGCTGTGCCTTCATG
B20fwd	CATGAAGGCACAGCCTGTTACA
R32rev	GATACCTACTACTGCGCGCATTTA
B18 rev	CGTATAGGAATTGCGAGCTTGA
B18fwd	TCAAGCTCGCAATTCCTATACG
R11rev	ACCCCGTATCCTATCACGTGTCA
R11fwd	TGACACGTGATAGGATACGGGGT
