# Supplementary material for: Sequence Analysis of microRNAs Encoded by Simian Lymphocryptoviruses
Source: Viruses. 2024 Dec 16;16(12):1923. doi: 10.3390/v16121923 (PMC11680086; doi:10.3390/v16121923)
Supplement: Supplementary file 1 [file viruses-16-01923-s001.zip › viruses-3351100-supplementary/Table S3 CeHV12 mIRs.pdf]

Table S3. CeHV12 miRNA sequences, S594 cells

Pre-miR	5p	3p	Read Counts (5p, 3p)
1	TAACCTCTCCTGGCACCCGAATT	CCCGGTTCCAGCAGAGGTTAG	764, 7021
2	AAATTTTGCCGTAGCAGATAGT	TATCTTTTGCGGTGAAATTTGA	5034, 675
3	TGACAGACAGTAAGGAGTGCGTC	TGCCCTTCGCACTGTCTGTTGGA	10987, 289432
4	TAGATGACAGGGGGGGCACAGT	TGGCCTCTACACGTCATCTTAAT	685, 51
5	TCAAGCTCGCAATTCCTATACG	TATAGGAATAATGAACTTGGTA	16266, 110264
6	ACCTATCCTATTGTGTTTTCACG	TGACACGTGATAGGATACG	95, 5668
7	AACCAGCTAACGCACAGTTGGA	AACGGTGCATGAACTGGTTACT	27, 8843
8	CCTCGGGCCCTCACTATGTTGC	CCACATAGAATGAGCTCGGGTTT	5571, 74852
9	TGCTGAACCCGGAATTGAACACA	TCACAATTCAAGGGTTTGGTAGC	1323, 29432
10	AAACCGTCTCTTGAGTTATGTG	TACATAACTGAGGAGAGCGGTTT	1641, 962
11	ACCCGCCCCTCACGACCGGGC	TCCTGTAGTGTGTGGTGTGGTGT	16, 27357
12	TAGAAGGCGTAACTTTCCTTGTGCT	CAGGAATGCTCCGCCTTCTAGA	20829, 1431
13	CCATTCGCTTTAAAACAAAACAT	TTTTGTTTAAAGCCAATGTCT	264, 3834
14	TAGCAGGCATGTCTTCATTCT	CATGAAGGCACAGCCTGTTACA	230, 1109
15	TACCTGTCCTTTGCAATCGGA	TTCGTTGCAAAGGTGAGGTGCT	52139, 1893
16	TAACTTACAACTGCCTGTTTGCA	TAAATGCGCGCAGTAGTAGGTATC	1650, 4126
17	?	TAGCACCACTATGCACTGTGTCT	?, 8273
18	GAAGGTGAACATAGCTGCCCATC	GTGGGCCGCTGTTCACCTCT	6605, 230
19	GGAGGGAAGCAGCGGCCAACTG	ATCAGTGGGCCTGTTTCCCTT	254, 75
